# Intermediate Monocytes and Circulating Endothelial Cells: Interplay with Severity of Atherosclerosis in Patients with Coronary Artery Disease and Type 2 Diabetes Mellitus

**DOI:** 10.3390/biomedicines11112911

**Published:** 2023-10-27

**Authors:** Irina V. Kologrivova, Tatiana E. Suslova, Olga A. Koshelskaya, Elena S. Kravchenko, Olga A. Kharitonova, Ekaterina A. Romanova, Alexandra I. Vyrostkova, Alla A. Boshchenko

**Affiliations:** 1 Cardiology Research Institute, Tomsk National Research Medical Center, Russian Academy of Sciences, 111A Kievskaya, Tomsk 634012, Russia; tes@cardio-tomsk.ru (T.E.S.); koshel@live.ru (O.A.K.); nikonova@cardio-tomsk.ru (E.S.K.); hoa@cardio-tomsk.ru (O.A.K.); bosh@cardio-tomsk.ru (A.A.B.); 2 Department of Biomedicine, Siberian State Medical University, 2 Moskovskii trakt, Tomsk 634050, Russia; katyromanova24@mail.ru (E.A.R.); alexandra.vy20@gmail.com (A.I.V.)

**Keywords:** atherosclerosis, monocytes, circulating endothelial cells, endothelial progenitor cells, coronary artery disease, type 2 diabetes mellitus, TGF-beta

## Abstract

The aim was to investigate the association of monocyte heterogeneity and presence of circulating endothelial cells with the severity of coronary atherosclerosis in patients with coronary artery disease (CAD) and type 2 diabetes mellitus (T2DM). We recruited 62 patients with CAD, including 22 patients with DM2. The severity of atherosclerosis was evaluated using Gensini Score. Numbers of classical (CD14++CD16–), intermediate (CD14++CD16+), and non-classical (CD14+CD16++) monocyte subsets; circulating endothelial progenitor cells; and the presence of circulating endothelial cells were evaluated. Counts and frequencies of intermediate monocytes, but not glycaemia parameters, were associated with the severity of atherosclerosis in diabetic CAD patients (r_s_ = 0.689; *p* = 0.001 and r_s_ = 0.632; *p* = 0.002, respectively). Frequency of Tie2+ cells was lower in classical than in non-classical monocytes in CAD patients (*p* = 0.007), while in patients with association of CAD and T2DM, differences between Tie2+ monocytes subsets disappeared (*p* = 0.080). Circulating endothelial cells were determined in 100% of CAD+T2DM patients, and counts of CD14++CD16+ monocytes and concentration of TGF-β predicted the presence of circulating endothelial cells (sensitivity 92.3%; specificity 90.9%; AUC = 0.930). Thus, intermediate monocytes represent one of the key determinants of the appearance of circulating endothelial cells in all the patients with CAD, but are associated with the severity of atherosclerosis only in patients with association of CAD and T2DM.

## 1. Introduction

The presence of diabetes mellitus (DM) nearly doubles the risk of the development of cardiovascular complications [1]. Type 2 DM (T2DM) is the most widespread form of diabetes, associated with insulin resistance and relative insulin insufficiency. The projected morbidity of T2DM is 786 million patients worldwide by 2045 [2]. Pathophysiological links mediating an immensely close interconnection between T2DM and atherosclerosis include increased levels of pro-atherogenic lipids, hyperglycemia, oxidative stress, endothelial dysfunction, and increased inflammation [3,4]. Even though distinct therapeutic strategies directed to control either hyperglycemia or inflammation demonstrated successful results in the prevention of cardiovascular risk in T2DM patients [5,6], the issue of the specific management of patients with association of CAD and T2DM remains largely unresolved. This underscores the search of the definite cellular-molecular factors being involved in the pathogenesis of atherosclerosis in this group of patients.

Monocytes, constituting 3–8% of all white blood cells, are active players in the development of inflammation in the course of both atherosclerosis and T2DM and represent a circulatory pool for future intra-plaque macrophages [7,8]. Human monocytes are subdivided into three subsets based on the surface expression of molecules CD14 and CD16. The majority of the cells are represented by classical monocytes, expressing only CD14 (CD14++CD16– monocytes), followed by non-classical (express mainly CD16, CD14+CD16++) and intermediate monocytes (express both CD14 and CD16, CD14++CD16+ monocytes) [9]. Several clinical studies demonstrated the fact of elevated frequencies and counts of non-classical and intermediate monocytes in patients with cardiometabolic disorders [10,11]. However, mechanisms through which these linkages are mediated and T2DM implications into the revealed changes remain majorly vague.

Monocytes perform constant surveillance of the endothelium, and upon sensing danger signals associated with endothelial injury, classical monocytes extravasate into the subendothelial space with a subsequent differentiation into macrophages, while non-classical monocytes are attracted to the lesion site, supporting endothelial homeostasis [12,13]. Activation of endothelial cells in the course of inflammation predisposes to the development of atherosclerosis [14]. Circulating endothelial cells (CEC) appear in the blood after shedding from the vascular wall as a result of the vascular injury. On the contrary, endothelial progenitor cells (EPC) are mobilized from the bone marrow and are involved in vascular repair [15]. The number of these cells corresponds to the values of flow mediated vascular dilation. Hence, their detection in peripheral blood represents a promising non-invasive approach for vascular health evaluation [16]. Earlier studies have demonstrated that endothelial dysfunction may precede the development of morphological lesions in patients with T2DM for decades, and elevation of CECs accompanied by the decrease of EPCs was typical to T2DM patients with microvascular complications [17]. However, conventional methods of flow cytometry did not allow to reliably verify the endothelial phenotype of the cells, which led to some inconsistency in the results. The approaches in flow cytometry involving the option of visualization made validation of CECs and EPCs morphology easier and allowed us to obtain more conclusive information [18]. However, modern data on the CECs and EPCs in patients with association of stable CAD and T2DM are absent, and the impact of monocyte subsets on the mobilization of circulating cells of endothelial nature has not been evaluated.

In the present study, we aimed to investigate monocyte heterogeneity and the presence of circulating endothelial cells with respect to the severity of coronary atherosclerosis in patients with association of coronary artery disease (CAD) and T2DM. Both specific cellular populations and concentration of soluble biomarkers potentially involved in the polarization of monocytes and development of endothelial dysfunction were analyzed.

## 2. Materials and Methods

### 2.1. Patients

An observational, single-center, cross-sectional, comparative study was performed. We have enrolled 40 patients with stable chronic CAD (group 1) and 22 patients with stable CAD associated with T2DM (group 2). Only patients scheduled for selective coronary angiography were enrolled to verify the presence/absence and the severity of atherosclerosis.

The study was conducted in accordance with the guidelines of the Declaration of Helsinki with amendments as of 2000 and “Rules of Clinical Practice in the Russian Federation”, approved by the Order of the Ministry of Health of the Russian Federation on 19 June 2003 No. 266. The protocol of the study was approved by the Biomedical Ethics Committee of Cardiology Research Institute, Tomsk NRMC (protocol No. 210 from 18 February 2021). All the individuals recruited to the study provided their informed consent. Diagnosis of T2DM was established according to the criteria of the modern classification of diabetes mellitus [1].

Exclusion criteria were as follows: body weight fluctuations higher than 3% during the last 3 months; complications of CAD less than 6 months prior to the enrollment into the study; any active inflammatory process other than atherosclerosis; history of any infectious diseases, current or recurrent; chronic kidney disease more severe than G3b-class according to CGA staging; autoimmune and autoinflammatory conditions; blood diseases; cancer; refusal to take part in the study.

Anthropometry included measurement of patients’ height and weight with the subsequent calculation of body mass index (BMI). The degree of abdominal obesity was evaluated according to the size of the waist circumference.

All the patients underwent selective coronary angiography on angiographic complex Cardioscop-V and computer system Digitron-3NAC, Siemens (Munich, Germany). The severity of atherosclerosis was estimated via calculation of Gensini Score [19].

### 2.2. Blood Processing

Fasting peripheral venous blood was obtained from all the patients in EDTA tubes and clotting activator tubes at least 5 days after coronary angiography. Blood was additionally drawn 2 h after the standard meal in clotting activator tubes for evaluation of postprandial glucose. Clotting activator tubes were centrifuged at 1500× *g*, and blood serum was aliquoted in plastic tubes and stored at −40 °C until the final processing.

### 2.3. Flow Cytometry

Peripheral blood mononuclear leucocytes (PBMC) were obtained from EDTA blood via centrifuging with Histopaque 1077 (Sigma, Livonia, MI, USA). We identified monocytes subsets using the following combination of monoclonal antibodies conjugated with corresponding fluorochromes: anti-CD14-Phycoerythrin (PE), anti-CD16-Fluorescein isothiocyanate (FITC), anti-HLA-DR- Phycoerythrin-Cyanine5 (PE-Cy5) (all reagents: BD Biosciences, San Jose, CA 95131, USA). The percentages of classical CD14++CD16–, intermediate CD14++CD16+ and non-classical CD14+CD16++ monocytes were evaluated, and absolute counts of monocytes were calculated based on the results of flow cytometry and the routine complete blood count performed on the automatic hematological analyzer. In several patients, monoclonal antibodies to tunica intima endothelial kinase 2 (Tie2) conjugated to Alexa Fluor 647 (AF647) (BD Biosciences, San Jose, CA 95131, USA) were added to the panel. Staining of circulatory endothelial cells was performed using the following panel of monoclonal antibodies conjugated to fluorochromes: anti-CD106-FITC, anti-CD45-PE-Cy5, anti-CD146-AF647, anti-CD31-PE-Cy7 (BD Biosciences, San Jose, CA 95131, USA). Staining of endothelial progenitor cells was performed with the panel of monoclonal antibodies conjugated to fluorochromes: anti-CD45-FITC, anti-CD34-PE, anti-CD31-PE-Cy7, anti-CD133-Allophycocyanin (APC) (BD Biosciences, San Jose, CA 95131, USA).

**Figure 1:**
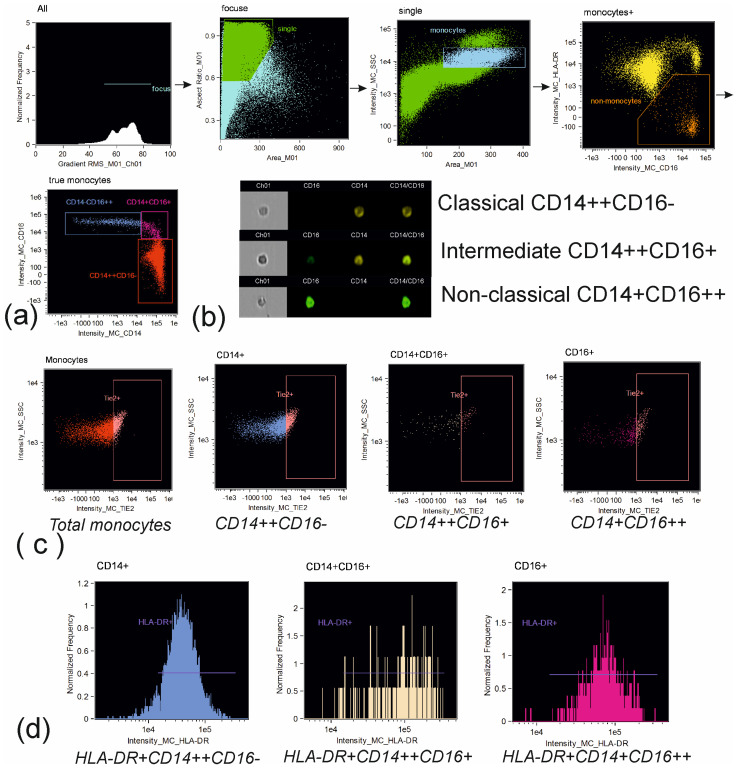
Gating strategy for monocytes subset: (**a**) gating of classical (CD14++CD16–), intermediate (CD14++CD16+) and non-classical (CD14+CD16++) monocytes: identification of cells in focus; single cells; monocytes via parameters of area and side scatter; exclusion of CD16+HLA-DR– cells; identification of regions for monocytes subsets; (**b**) representative images of classical (CD14++CD16–), intermediate (CD14++CD16+) and non-classical (CD14+CD16++) monocytes; (**c**) identification of Tie2+ monocytes; (**d**) identification of HLA-DR+ monocytes.

**Figure 2:**
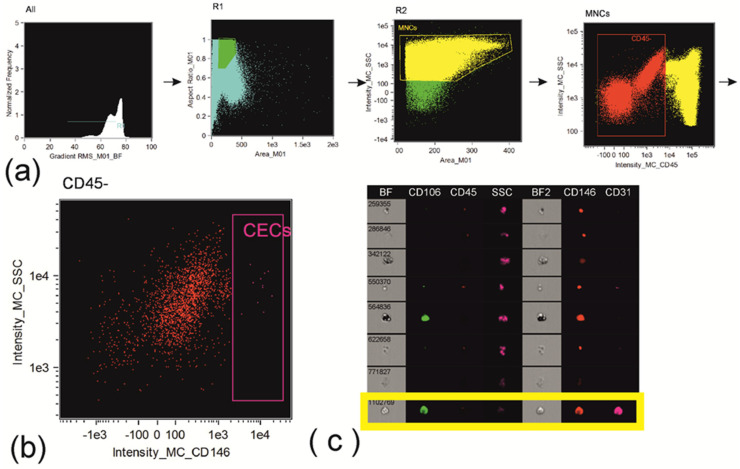
Gating strategy for circulating endothelial cells: (**a**) gating of CD45– cells: identification of cells in focus; single cells; peripheral blood mononuclear cells via parameters of area and side scatter; identification of CD45– cells; (**b**) gating of CD45–CD146+ cells; (**c**) verification of circulatory endothelial cells using image gallery—only one cell out of several gated cells represents circulatory endothelial cell based on cellular morphology and expression of specific endothelial markers.

**Figure 3:**
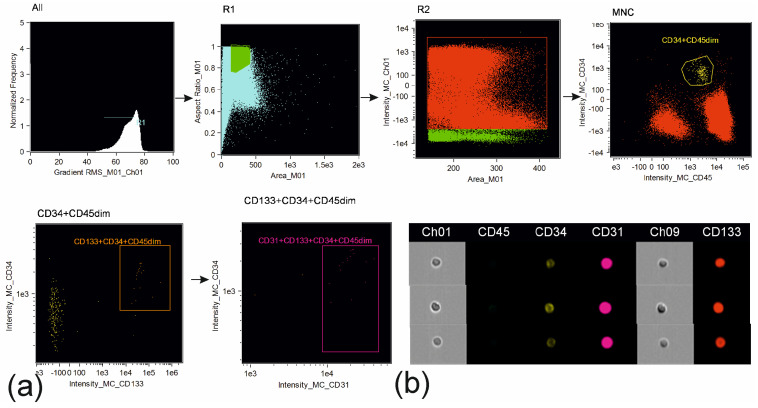
Gating strategy for endothelial progenitor cells: (**a**) gating of cells in focus; single cells; peripheral blood mononuclear cells via parameters of area and intensity of autofluorescence in brightfield channel; identification of CD34+CD45dim cells; identification of CD34+CD45dimCD133+ cells; identification of CD34+CD45dimCD133+CD31+ cells; (**b**) representative images of endothelial progenitor cells.

Cells were analyzed on an Amnis FlowSight (Cytek Biosciences, Fremont, CA, USA) instrument equipped with 488 nm and 642 nm lasers and INSPIRE software version 100.3.218.0 (Amnis Corporation, Seattle, DC, USA). Brightfield images were acquired on channel 1. Side-scatter was evaluated in channel 6 using 785 nm laser. Compensation matrix was created using compensation controls, representing samples stained with the singular fluorochrome conjugated to the monoclonal antibodies used in the research. The processing of the acquired data was performed in IDEAS 6.2.64.0 software (Amnis Corporation, Seattle, DC, USA). Compensated image files were created after application of compensation matrix to the raw files. At the first step of the analysis blurred cell images were excluded using histogram of “Gradient RMS” feature for images obtained in brightfield channel. At the second step of the analysis, cell doublets and large cell aggregates were excluded using the dot plot aria vs. aspect ratio of the brightfield images (representing ratio between the shortest and the longest axis of each event). The aspect ratio threshold equal to 0.6 and medium area of images were used to identify events to be included in the further analysis. Gating was verified using cell images from the image gallery (Figure 1, Figure 2 and Figure 3).

### 2.4. Multiplex Analysis of Cardiovascular Biomarkers

The MILLIPLEX map human cardiovascular panel was used to detect 4 cardiovascular biomarkers in total (endocan-1, FABP-3, FABP-4, PlGF) in blood serum using Multiplex Instrument FLEXMAP 3D Luminex Corporation and MILLIPLEX Analyst 5.1 software (Merck KGaA, Milliplex; Darmshdadt, Germany) at the Core Facility “Medical genomics”, Tomsk NRMC.

### 2.5. Enzyme-Linked Immunosorbent Assay (ELISA)

Concentrations of insulin, C-peptide (both—AccuBind kits, Diagnostic System Laboratories, Lake Forest, CA, USA), hsCRP, IL-1β (all cytokine kits—VECTOR-BEST, Novosibirsk, Russia), transforming growth factor-β (TGF-β) (Invitrogen, Vienna, Austria), insulin-like growth factor (IGF) (Mediagnost, Reutlingen, Germany), endothelin (Sigma, Darmstadt, Germany), proprotein convertase subtilisin/kexin type 9 (PCSK9) (R&D Systems, Bio-Techne, Minneapolis, MN, USA) were measured in serum using ELISA on an Infinite F500 (Tecan, Männedorf, Switzerland) instrument.

### 2.6. Biochemical Assays

Biochemical parameters (concentrations of glucose, glycated hemoglobin, total cholesterol, triacylglycerol (TG), high-density lipoprotein (HDL) cholesterol) were detected on a Cobas 6000 C501 (Roche, Mannheim, Germany) automatic analyzer. The Friedwald equation was used to calculate concentration of low-density lipoprotein (LDL) cholesterol: LDL (mM) = total cholesterol (mM) − (HDL (mM) + TG (mM)/2.2). Index TyG was calculated based on fasting concentrations of triglycerides and glucose using formula: ln (TG (mg/dL) × fasting glucose (mg/dL)/2) [20].

### 2.7. Statistical Analysis

Assessment of sample data distribution was performed using the Shapiro–Wilk test. Continuous variables were represented as medians and interquartile intervals (Q_1_; Q_3_). Nominal variables were represented as absolute numbers (n) and percentages (%). The Kruskal–Wallis ANOVA test was used for assessment of the differences between multiple independent groups. Differences between independent groups were analyzed using the Mann–Whitney U test with Bonferroni correction for multiple comparisons. Differences between the nominal variables were analyzed using Pearson’s χ^2^ test or Fisher Exact test. Analysis of the correlations between the variables was performed using the Spearman correlation coefficient. The prognostic significance of variables, predicting appearance of the circulating endothelial cells was evaluated by multiple logistics regression. All the statistical analysis was performed in Statistica 10.0 (StatSoft Inc., Tulsa, OK, USA). Two-sided *p* value < 0.05 was considered significant.

## 3. Results

### 3.1. Baseline Characteristics of Patients

The group of patients with T2DM included more women compared to the group of CAD patients without diabetes. The severity of atherosclerosis based on the Gensini Score and frequency of patients with anatomic stenosis >50% and >70% of one or several main coronary arteries (trunk, anterior descending artery, circumflex artery, right coronary artery) were comparable between groups (Table 1). As expected, recruited patients with CAD+T2DM received insulin and oral hypoglycemic drugs more frequently compared to non-diabetic CAD patients (Table 1).

### 3.2. Metabolic Parameters

Even though the prescribed medication allowed to achieve nearly all target values of glycemia in the majority of diabetic patients [1], patients with CAD+T2DM still were characterized by higher fasting and postprandial glucose, glycated hemoglobin, and TyG index levels compared to non-diabetic CAD (Table 2). CAD patients had the highest levels of PCSK9 compared to patients without T2DM (Table 2).

### 3.3. Monocytes Subsets

The frequency of intermediate CD14++CD16+ monocytes was higher in CAD+T2DM compared to CAD patients. However, these differences did not reach the level of statistical significance (Table 3).

### 3.4. Expression of Activation Markers on Monocytes Subsets

We evaluated expression of the cellular activation marker HLA-DR on monocyte subsets. It appeared to approach 100% and was comparable in patients from both groups (Table 4).

### 3.5. Expression of Tie2 on Monocyte Subsets in CAD Patients

**Figure 4:**
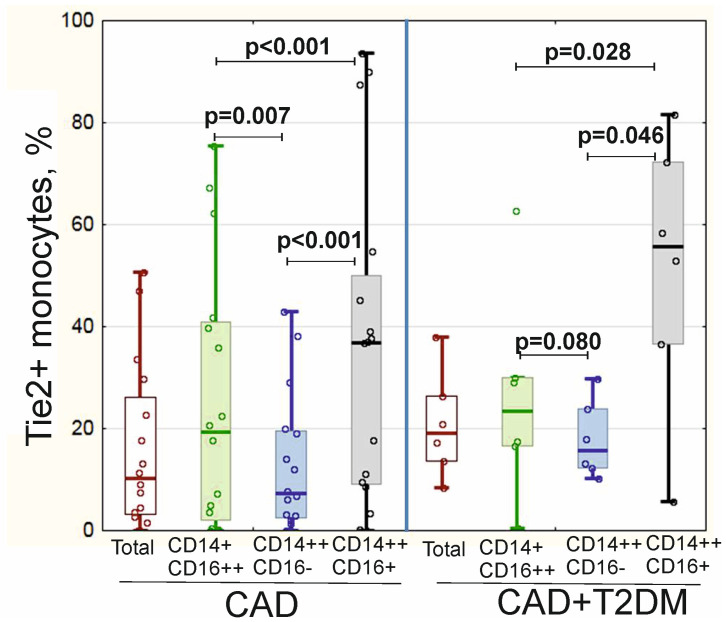
Tie2+ expression on monocyte subsets in patients with coronary artery disease depending on the presence of type 2 diabetes mellitus.

Intermediate CD14++CD16+ monocytes expressed proangiogenic marker Tie2+ with the highest frequency compared to other monocyte subsets in patients both with and without T2DM (Figure 4). Presence of T2DM impacted distribution of Tie2 between classical and non-classical monocytes: frequency of Tie2+ cells was lower in classical compared to non-classical monocytes in CAD patients, while in patients with association of CAD and T2DM significant differences between Tie2+ monocytes subsets were absent (Figure 4).

### 3.6. Circulating Endothelial and Endothelial Progenitor Cells

**Figure 5:**
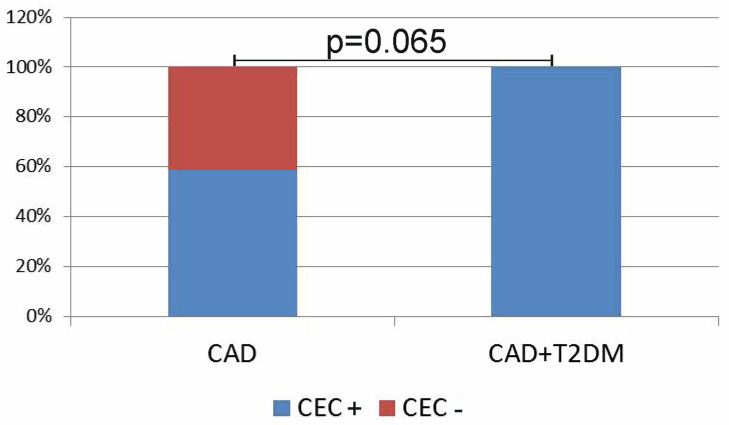
Presence of circulating endothelial cells in groups of patients.

CECs were detected in all the patients with association of CAD and T2DM, while in patients with CAD, CECs were detected only in 60% (Figure 5). Even though the differences did not reach the level of statistical significance, we observed an obvious tendency of T2DM to predispose to the appearance of CECs in the peripheral blood.

Patients with association of CAD and T2DM had lower frequencies and absolute counts of endothelial progenitor cells compared to CAD patients without T2DM. However, with differences also not reaching the level of statistical significance (Table 5).

### 3.7. Biomarkers of Inflammation and Endothelial Dysfunction

In patients with association of CAD and T2DM concentration of FABP-3 tended to be higher in CAD patients with T2DM compared to nondiabetic CAD patients (Table 6). Other biomarkers were comparable between groups of patients (Table 6).

### 3.8. Association of CECs with Monocyte Subsets

We have performed multiple logistic regression analysis in the total group of patients to evaluate the significant predictors of CEC appearance in circulation. It turned out that absolute count of intermediate CD14++CD16+ monocytes and concentration of TGF-β allowed prediction of the presence of CEC in patients (Table 7).

**Figure 6:**
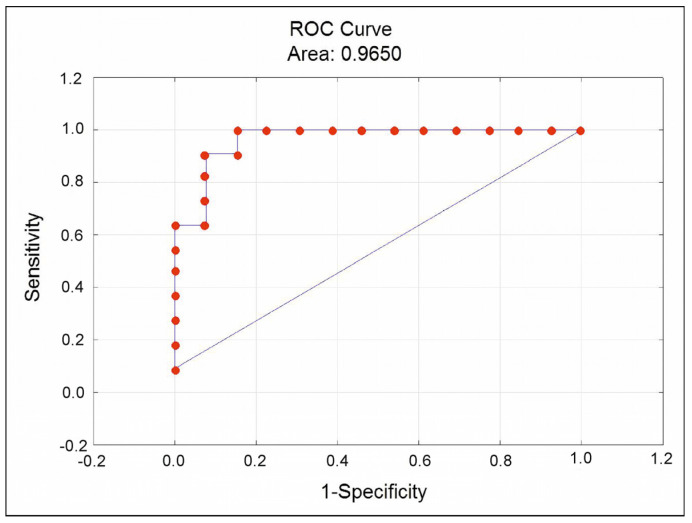
ROC curve of the multiple logistics regression for classification of patients into groups with presence or absence of circulating endothelial cells.

The significance level of the model was *p* = 0.0000149. The prognostic characteristics of the model were as follows: sensitivity 92.3%, specificity 90.9%, AUC = 0.930. ROC-curve is represented in Figure 6. The cutoff level of the probability of CEC appearance was 0.596.

### 3.9. Factors Influencing the Severity of Atherosclerosis in CAD and CAD+T2DM Patients

In CAD patients without T2DM index, Gensini Score—reflecting the severity of atherosclerosis—was associated with metabolic biomarkers, such as fasting glucose, concentration of heart-type FABP-3, and concentration of endothelial dysfunction biomarkers—endocan-1 and placental growth factor (PlGF), a cytokine stimulating angiogenesis and inducing atherosclerosis (Table 8). Concentrations of HDL cholesterol and IGF displayed negative associations with the severity of atherosclerosis in CAD patients (Table 8).

In patients with association of CAD and T2DM the main factors associated with the severity of atherosclerosis included absolute counts and frequencies of intermediate CD14++CD16+ monocytes and concentration of FABP-3 (Table 8). Of note, in diabetic patients, unlike patients with CAD only, FABP-3 was inversely associated with Gensini Score. There was also revealed tendency for endocan-1 to be associated with the severity of atherosclerosis in CAD+T2DM patients (Table 8).

Only in patients with presence of endothelial cells Gensini Score correlated with the frequency of Tie2+ cells in total monocytes’ subset (r_s_ = 0.664; *p* = 0.018), Tie2+CD14+CD16++ monocytes (r_s_ = 0.478; *p* = 0.005) and Tie2+CD14++CD16– monocytes (r_s_ = 0.601; *p* = 0.039).

## 4. Discussion

In our work we demonstrated new cellular–molecular features typical for patients with association of CAD and T2DM. In particular, we demonstrated that intermediate monocytes are associated with the severity of atherosclerosis in these patients and, presumably, are involved in the process of vascular injury, as intermediate monocytes together with TGF-β were the main determinants of the appearance of CECs in peripheral blood.

Intermediate monocytes are absent in mice and constitute 2–11% of all monocytes in humans. There is a hypothesis that they represent a transitional stage in the differentiation of non-classical monocytes from classical monocytes. On the other hand, non-classical and intermediate monocytes were demonstrated to possess a distinct phenotype [21]. Intermediate monocytes were elevated in the course of cardio–vascular diseases, cancer, stroke, various autoimmune disorders, bacterial and viral infections [22] and were attributed specific functions, such as secretion of inflammatory cytokines and chemotaxis into tissues due to the expression of chemokine receptors. Since mice are missing intermediate monocyte subsets, the results obtained on monocytes subsets in animal models of atherosclerosis cannot be fully extrapolated into humans. However, intermediate monocytes were already related to predisposition to atherosclerosis [23], were increased in patients with stable CAD compared to healthy controls [24,25], and were elevated in acute cardiovascular conditions [26]. Ivo N. SahBandar et al. (2020) have revealed that absolute count of intermediate monocytes directly correlated to intima-media thickness of right common carotid artery and right carotid bifurcation in the general population [27]. Patients with stable angina and concentration of Lp(a) above 50 mg/dL, a known risk factor for the development of atherosclerosis, were characterized by the increased frequency of intermediate monocytes compared to those with Lp(a) below 50 mg/dL [28]. In patients with stable angina cells expressing both CD14 and CD16 have been demonstrated to be associated with the development of multi-vessel disease rather than single-vessel and correlated to the values of Gensini Score. However, the influence of T2DM was not analyzed in this study even though T2DM patients were recruited, and the authors did not separate the subsets of non-classical and intermediate monocytes in their study [29]. Meanwhile, we revealed interconnection of intermediate monocyte with the severity of atherosclerosis only in CAD patients with T2DM, while in non-diabetic CAD patients, there were other non-cellular biomarkers associated with a degree of Gensini Score.

T2DM is also known to impact skewing of monocytes subsets [8]. Monocytes expressing angiopoietin receptor tunica intima endothelial kinase 2 (Tie2+ monocytes) were originally found in tumors, and their primary function was identified as promotion of pathological tumor angiogenesis [30]. The presence of Tie2+ monocytes has recently been reported in T2DM patients [31] and in patients with critical limb ischemia [32]. Reijrink M. et al. (2022) have demonstrated increased frequencies of Tie2+ intermediate monocytes among CAD patients with T2DM and decreases in non-classical monocytes [8]. We did not reveal significant differences in Tie2+ intermediate monocytes frequencies between patients with and without T2DM but rather observed a disruption between Tie2 expression on classical/non-classical monocytes, which could have contributed to the inability for successful endothelium surveillance by monocytes. Interestingly, in proatherogenic environments the functional discrepancies between monocytes subsets became blurred, and all three subsets possessed proinflammatory properties—for example, an increase in cytokine production [7], which may partially explain controversial data on the role of monocytes in atherogenesis.

There are several mechanisms of atherosclerosis progression in diabetic patients involving monocytes/macrophages, such as elevated recruitment into the vascular wall, increased monocyte activation, impairment of lipid uptake and storage, increased susceptibility to cellular death, and inefficient efferocytosis [33]. There is also evidence that the proatherogenic activity of monocytes lies far beyond their potential to further differentiate into plaque macrophages and includes crosstalk with the endothelium [34,35].

CECs may appear in circulation when the endothelium loses its integrity in the process of continuous exposure to cardiovascular risk factors, including inflammation [36]. Hyperglycemia is known to unfavorably influence integrity of the endothelial vascular layer, as it leads to increased production of reactive oxygen species, DNA damage, and elevated permeability of the endothelium [37], and intermediate monocytes may be involved in the mediation of these processes according to our results.

There is no solid answer to the question of whether impairment of the integrity of endothelial monolayer is caused by hyperactivation of intermediate monocytes or exposure to subendothelial space—leading to the appearance of danger associated molecular patterns (DAMPs)—leads to overproduction of intermediate monocytes [14]. In any case, monocytes and macrophages have been demonstrated to play an active part in the sustenance of the integrity of endothelium and preservation of its function. In early works devoted to vascular collateral regeneration, accumulation of monocytes was observed in the area of collateral arteries following modeled ischemia, which indicates input of monocytes in arterial remodeling, underscoring their protective properties [12].

There are several possible mechanisms through which interaction between intermediate monocytes and endothelial cells may take place. First, monocytes may potentiate expression of adhesion molecules on the surface of endothelial cells. Second, communication between monocytes and endothelial cells may be mediated by extracellular vesicles (EVs) [38]. Micro-RNAs contained in endothelial EVs may both activate and suppress monocytes and macrophages [39,40]. This interconnection is reciprocal, as monocyte-derived EVs may also influence endothelial cells, promoting their apoptosis and reducing EC proliferation [41,42].

Interestingly, notwithstanding the mainly anti-inflammatory effects of TGF-β in other tissues, this growth factor has recommended itself as a potent pro-inflammatory regulator when it comes to its function in endothelial cells. Inhibition of its endothelial signaling in hyperlipidemic mice was associated with a reduction of vascular inflammation and permeability and led to a decrease in the progression of atherosclerosis [43]. TGF-β has been shown to favor endothelial cells’ trans-differentiation into mesenchymal cells [43]. This explains TGF-β being a significant determinant together with a frequency of intermediate monocyte in the appearance of CECs in our study.

Elevation of CECs without concomitant elevation of EPCs in group of T2DM patients represents an unfavorable sign of vascular injury without sufficient potential of endothelial and vascular recovery [14]. Of note, even when present in circulation, EPCs are very unlikely to participate in the repair of the endothelium, which downplays the importance of their implementation in regenerative medicine [44,45]. Some other approaches—probably involving the targeting of other cells, such as monocytes—are required to guarantee the maintenance of endothelium integrity.

We have revealed the association of the severity of atherosclerosis with serum concentration of endocan-1 both in the groups of patients with and without T2DM even though in T2DM patients the interconnection with endocan-1 was less significant (Table 8). Endocan-1 or endothelial-cell-specific molecule-1 is secreted by endothelial cells and represents a marker of endothelial activation during CAD [46]. Its production is stimulated by inflammatory mediators and is inhibited by interferon (IFN)-γ [47]. The function of endocan-1 remains poorly understood, but most likely, increases in its production are associated with activation of the endothelium and increased production of soluble intercellular adhesion molecule-1 (sICAM-1) and soluble vascular cell adhesion molecule-1 (sVCAM-1) [48]. Our results and the results of other researchers [49] indicate that levels of endocan-1 are associated with the development of atherosclerosis. Even though previous studies have revealed overexpression of endocan-1 in the serum of T2DM patients [50], we did not find any differences in endocan-1 concentration depending on diabetic status. One of the possible explanations is statin intake in the majority of patients, as rosuvastatin in the dose 40 mg was demonstrated to significantly reduce endocan levels in blood [51].

Inverse correlation between IGF concentration and the severity of atherosclerosis was revealed only in patients without T2DM. IGF is known to reduce infiltration of macrophages into atherosclerotic lesion and expression of pro-inflammatory cytokines, thus mediating anti-inflammatory and anti-atherosclerotic effects. IGF was also demonstrated to increase numbers and to enhance the properties of endothelial progenitor cells [52] and promoted polarization of monocytes towards an inflammatory phenotype in a murine model of colitis [53].

FABP-3 is a carrier protein of polyunsaturated fatty acids (PUFAs) and is expressed mainly in the heart, skeletal muscles, and the kidneys [54]. Recently it was also shown to possess immune properties, potentiating B-cell-mediated effects [55]. Macrophages, myeloid dendritic cells, and endothelial cells appeared to be capable of FABP-3 production in pro-inflammatory conditions, typical for atherosclerosis [56,57]. In these circumstances, the effects of FABP-3 on the endothelium were detrimental: exogenous FABP-3 increased LPS-induced inflammation, while silencing of FABP-3 in endothelial cell culture promoted protective, anti-inflammatory, and pro-angiogenic effects [57]. FABP-3 also played a role in cholesterol uptake by macrophages and in the formation of foam cells, thus exhibiting pro-atherogenic effects [58]. Thus, indirect relationships between FABP-3 levels and Gensini Score in diabetic patients rather indicate that other pathophysiological pathways acquire higher importance in the progression of atherosclerosis in this patients’ cohort.

Surprisingly, in patients with association of CAD and T2DM, the severity of atherosclerosis did not depend on the levels of fasting glycemia, unlike patients with CAD only. This may be partially due to the fact that CAD+T2DM patients received glucose-lowering therapies at higher frequencies than CAD patients (Table 1). However, the received medication obviously was not sufficient to modify associations between numbers of intermediate monocytes and severity of atherosclerosis. Since the surrogate index of insulin resistance TyG was elevated in diabetic CAD patients, one may suspect that quality of the combined triglycerides and glycaemia control may impact the severity of atherosclerosis in these patients. New glucose- and lipid-lowering strategies need to be introduced in regular practice in future keeping in mind their potential impact on composition of monocytes subsets, especially on the counts of intermediate monocytes. Supplementation with omega-3 polyunsaturated acids in general reduced inflammation and normalized triglycerides’ levels, but responders and non-responders to omega-3 differed with respect to gene transcripts related to sphingolipid metabolism in peripheral blood mononuclear leucocytes [59], which underscores the importance of a personalized therapeutic approach. Both fibrates and statins downregulated production of inflammatory cytokines by monocytes independently from the lipid-lowering effect [7]. However, in some studies, statins were not sufficient to reverse proinflammatory changes in monocytes at the epigenetic level [60]. It is worth noting that in our study, the majority of patients were taking statins in suboptimal doses due to the developed side effects or achievement of the maximum tolerated dosage of statins without reaching the target LDL values. Further research needs to be conducted to the evaluate dose-dependent impact of statins on monocyte subsets and the condition of the endothelium.

Limitations of the present study include its small size and cross-sectional design. An increase in the number of patients in the cohort of patients with association of CAD and T2DM could have allowed us to obtain statistically significant results in those analyses in which we revealed only tendencies. Additionally, functional assays targeting interaction between intermediate monocytes and properties of endothelial cells would have benefited understanding of the intricate crosstalk between these two cellular subsets. Another limitation is that all the cell evaluations were performed after diagnostic coronary angiography, which—despite being performed at least 5 days prior to analysis—represented an invasive procedure and could have caused a definite cell-mediated response [61]. Still, the fact that all the recruited patients similarly underwent this diagnostic procedure partially counterbalances the possible implications of it to the obtained results.

At the same time, our results indicate the existence of substantial differences in the cellular–molecular moiety involved in the progression of atherosclerosis in patients with and without diabetes. Prospective analysis in the future will allow us to distinguish cellular and humoral biomarkers which significantly impact the prognosis in patients with association of CAD and T2DM.

## 5. Conclusions

Thus, we have demonstrated for the first time that numbers of intermediate monocytes and concentration of TGF-β represent the main factors associated with the appearance of circulating endothelial cells, which reflect endothelial dysfunction and were present in all the patients with association of coronary artery disease and type 2 diabetes mellitus. Only in patients with type 2 diabetes mellitus were counts of intermediate monocytes correlated with the severity of atherosclerosis.

## Figures and Tables

**Table 1:** Baseline characteristics of patients.

Parameters	Group 1: Patients with CAD (n = 40)	Group 2: Patients with CAD+T2DM (n = 22)	*p*
Gender (m/f)	28/12	8/14	***p* = 0.015**
Age, years	64.0 (57.5; 66.0)	65.5 (62.0; 68.0)	*p* = 0.067
History of myocardial infarction, n (%)	12 (30.8)	10 (47.6)	*p* = 0.272
Arterial hypertension, n (%)	37 (94.8)	22 (100)	*p* = 0.546
Duration of diabetes, years	-	10.0 (3.0; 12.0)	-
Duration of coronary artery disease, years	2.5 (1.0; 8.0)	5.0 (3.0; 9.0)	*p* = 0.075
Systolic blood pressure, mm Hg	124.0 (116.0; 135.0)	134.5 (115.0; 140.0)	*p* = 0.229
Diastolic blood pressure, mm Hg	74.0 (65.9; 80.0)	70.0 (65.0; 78.0)	*p* = 0.196
Smoking, n (%)	18 (46.2)	4 (18.2)	*p* = 0.052
Body Mass Index, kg/m^2^	29.0 (26.2; 31.9)	31.2 (28.4; 34.6)	*p* = 0.051
Waist circumference, cm	100.0 (96.0; 111.0)	106.0 (99.0; 115.0)	*p* = 0.195
Stenosis > 50%, n (%)	31 (77.5)	18 (81.8)	*p* = 0.756
Stenosis > 70%, n (%)	25 (62.5)	17 (77.3)	*p* = 0.270
Gensini Score, points	32.8 (9.0; 59.5)	26.0 (15.0; 53.0)	*p* = 0.845
Oral hypoglycemic drugs, n (%)	2 (5.1)	20 (90.9)	***p* < 0.001**
Insulin, n (%)	0	6 (27.3)	***p* = 0.001**
RAAS inhibitors, n (%)	31 (79.5)	20 (95.2)	*p* = 0.300
Calcium channels antagonists, n (%)	21 (53.9)	12 (57.2)	*p* = 0.999
Beta-blockers, n (%)	30 (76.9)	17 (81.0)	*p* = 0.999
Diuretics, n (%)	12 (30.8)	9 (42.3)	*p* = 0.413
Statins, n (%)	34 (85.0)	19 (90.5)	*p* = 0.999

*p*—indicates the significance level of differences; Gensini Score represents a cumulative coronary atherosclerosis burden based on the results of coronary angiography; bold font indicates significant differences.

**Table 2:** Metabolic parameters of recruited patients.

Parameters	Group 1: Patients with CAD (n = 40)	Group 2: Patients with CAD+T2DM (n = 22)	*p*
Fasting glucose, mM	5.3 (5.0; 5.8)	7.1 (6.0; 7.9)	***p* < 0.001**
Postprandial glucose, mM	6.0 (5.4; 7.0)	9.3 (8.5; 14.1)	***p* < 0.001**
Glycated hemoglobin, %	6.0 (5.7; 6.5)	7.4 (6.5; 8.4)	***p* < 0.001**
Fasting insulin, µIU/mL	5.7 (2.4; 8.8)	4.3 (3.3; 7.7)	*p* = 0.901
Fasting C-peptide, ng/mL	2.8 (2.1; 3.3)	2.6 (2.0; 3.2)	*p* = 0.625
Total cholesterol, mM	4.1 (3.3; 5.2)	3.6 (2.9; 5.0)	*p* = 0.407
Triglycerides, mM	1.4 (1.1; 2.1)	1.8 (1.2; 2.2)	*p* = 0.391
HDL cholesterol, mM	1.1 (0.9; 1.2)	1.0 (0.9; 1.3)	*p* = 0.972
LDL cholesterol, mM	2.4 (1.8; 3.1)	2.0 (1.3; 2.9)	*p* = 0.224
TyG	3.8 (3.6; 4.0)	4.0 (3.9; 4.1)	***p* = 0.010**
PCSK9, ng/mL	257.9 (199.3; 274.7)	176.0 (150.9; 235.1)	***p* = 0.021**

*p*—indicates the significance level of differences; bold font indicates significant differences.

**Table 3:** Monocyte subsets in recruited patients.

Parameters	Group 1: Patients with CAD (n = 39)	Group 2: Patients with CAD+T2DM (n = 21)	*p*
CD14++CD16–, %	85.5 (79.5; 90.7)	85.8 (82.4; 88.2)	*p* = 0.735
CD14++CD16+, %	5.5 (3.5; 7.5)	6.4 (3.8; 7.9)	*p* = 0.498
CD14+CD16++, %	8.5 (5.2; 13.1)	8.6 (5.2; 9.2)	*p* = 0.432
CD14++CD16–, ×10^9^/L	46.9 (34.9; 56.5)	48.6 (42.1; 64.0)	*p* = 0.177
CD14++CD16+, ×10^9^/L	2.9 (1.9; 4.9)	3.7 (2.4; 5.4)	*p* = 0.149
CD14+CD16++, ×10^9^/L	4.3 (2.3; 5.8)	4.7 (3.3; 6.4)	*p* = 0.484

*p*—indicates the significance level of differences.

**Table 4:** Expression of HLA-DR in monocyte subsets.

Parameters	Group 1: Patients with CAD (n = 27)	Group 2: Patients with CAD+T2DM (n = 13)	*p*
CD14++CD16–HLA-DR+, %	89.7 (66.1; 93.8)	75.2 (64.0; 86.7)	*p* = 0.458
CD14++CD16+HLA-DR, %	97.8 (98.3; 100)	95.0 (92.2; 100)	*p* = 0.127
CD14+CD16++HLA-DR+, %	99.6 (98.3; 100)	100 (99.2; 100)	*p* = 0.331

*p*—indicates the significance level of differences.

**Table 5:** Endothelial progenitor cells in recruited patients.

Parameters	Group 1: Patients with CAD (n = 18)	Group 2: Patients with CAD+T2DM (n = 5)	*p*
Endothelial progenitor cells, % of PBMC	0.004 (0.002; 0.010)	0.003 (0.001; 0.008)	*p* = 0.745
Endothelial progenitor cells, ×10^6^/L	0.11 (0.04; 0.21)	0.06 (0.03; 0.24)	*p* = 0.857

*p*—indicates the significance level of differences.

**Table 6:** Plasma concentration of cytokines, chemokines, growth factors and endothelial dysfunction biomarkers in recruited patients.

Parameters	Group 1: Patients with CAD (n = 37)	Group 2: Patients with CAD+T2DM (n = 22)	*p*
C-reactive protein, mg/mL	3.42 (2.11; 8.35)	4.15 (1.29; 6.84)	*p* = 0.862
Endocan-1, pg/mL	1902.0 (1463.0; 2596.0)	1985.0 (1887.0; 2546.0)	*p* = 0.281
FABP-3, pg/mL	2944.0 (2087.0; 3598.0)	3431.0 (3060.0; 4284)	***p* = 0.081**
FABP-4, ng/mL	20.40 (9.01; 32.94)	20.61 (15.61; 59.49)	*p* = 0.328
PlGF, pg/mL	4.05 (1.28; 8.80)	5.89 (2.62; 7.58)	*p* = 0.603
IL-1β, pg/mL	0.86 (0.53; 1.22)	0.75 (0.51; 0.99)	*p* = 0.254
TGF-β, ng/mL	30.38 (25.27; 36.60)	33.68 (28.99; 42.38)	*p* = 0.246
IGF, μg/mL	67.74 (53.76; 104.07)	76.93 (63.10; 101.85)	*p* = 0.729
Endothelin, fmol/mL	0.37 (0.33; 0.66)	0.43 (0.35; 0.83)	*p* = 0.320

*p*—indicates the significance level of differences; bold font indicates significant differences and tendencies.

**Table 7:** Estimates of the multiple logistics regression model and their significance levels.

Parameters	Estimate	*p*
Intercept	26.63	0.030
CD14++CD16+	−1.337	0.047
TGF-β	−0.639	0.041

**Table 8:** Correlations between molecular and cellular biomarkers and Gensini Score in CAD patients depending on the presence or absence of type 2 diabetes mellitus.

	Gensini Score			
	Group 1: Patients with CAD		Group 2: Patients with CAD+T2DM	
Parameters	r_s_	*p*	r_s_	*p*
Fasting glucose	0.338	**0.035**	−0.232	0.298
HDL Cholesterol	−0.394	**0.013**	−0.305	0.168
Endocan-1	0.529	**0.010**	0.489	**0.089**
FABP-3	0.440	**0.036**	−0.552	**0.049**
PlGF	0.517	**0.011**	0.061	0.844
IGF	−0.416	**0.012**	−0.065	0.786
CD14++CD16+, %	−0.199	0.222	0.632	**0.002**
CD14++CD16+, ×10^9^/L	−0.183	0.272	0.689	**0.001**

bold font indicates significant correlations and tendencies.

## Data Availability

The raw data are available from the corresponding author upon reasonable request.
